# Awareness is required for autonomic performance monitoring in instrumental learning: Evidence from cardiac activity

**DOI:** 10.1111/psyp.14047

**Published:** 2022-03-18

**Authors:** Lina I. Skora, James J. A. Livermore, Federica Nisini, Ryan B. Scott

**Affiliations:** ^1^ School of Psychology University of Sussex Brighton UK; ^2^ Sackler Centre for Consciousness Science University of Sussex Brighton UK; ^3^ Donders Institute for Brain, Cognition and Behaviour Radboud University Nijmegen The Netherlands; ^4^ Center for Economics and Neuroscience Universität Bonn Bonn Germany

**Keywords:** cardiac deceleration, consciousness, ECG, performance monitoring, unconscious instrumental conditioning

## Abstract

Performance monitoring is a vital aspect of successful learning and decision‐making. Performance errors are reflected in the autonomic nervous system, indicating the need for behavioral adjustment. As part of this response, errors cause a pronounced deceleration in heart rate, compared to correct decisions, and precede explicit awareness of stimulus–response outcome contingencies. However, it is unknown whether those signals are present and able to inform instrumental learning without stimulus awareness, where explicit performance monitoring is disabled. With mixed evidence for unconscious instrumental learning, determining the presence or absence of autonomic signatures of performance monitoring can shed light on its feasibility. Here, we employed an unconscious instrumental conditioning task, where successful learning is evidenced by increased approach responses to visually masked rewarding stimuli, and avoidance of punishing stimuli. An electrocardiogram (ECG) assessed cardiac activity throughout the learning process. Natural fluctuations of awareness under masking permitted us to contrast learning and cardiac deceleration for trials with, versus without, conscious stimulus awareness. Our results demonstrate that on trials where participants did not consciously perceive the stimulus, there was no differentiation in cardiac response between rewarding and punishing feedback, indicating an absence of performance monitoring. In contrast, consciously perceived stimuli elicited the expected error‐related deceleration. This result suggests that, in unconscious instrumental learning, the brain might be unable to acquire knowledge of stimulus values to guide correct instrumental choices. This evidence provides support for the notion that consciousness might be required for flexible adaptive behavior, and that this may be mediated through bodily signals.

## Abstract 2

Performance monitoring is vital to successful learning. We provide novel evidence that autonomic performance monitoring, indexed by cardiac deceleration, is only engaged in conscious, but not unconscious, instrumental learning. This result provides support for the notion that consciousness is required for flexible adaptive behaviour, and that this relationship may be mediated by bodily signals.

## 1 INTRODUCTION

Performance monitoring is a critical aspect of successful learning and decision‐making. Efficient monitoring involves the ability to swiftly detect errors in performance in order to adjust future behavior accordingly. This can be achieved by monitoring external events for feedback—for example, the consequences of actions, such as reward or punishment. In the instrumental conditioning or learning process, actual outcomes of one's actions are compared to expected outcomes, with mismatches (e.g., following from an error) informing the learner of the necessity for behavioral adaptation. It has been proposed that performance monitoring is also reflected in the autonomic nervous system (ANS) (Hajcak et al., 2003; Müller et al., 2005; Sokolov, 1963; Ullsperger et al., 2014). Correlates of error‐ and feedback‐related activity have been observed in error‐related negativity (ERN or Ne) and positivity (Pe) components in electroencephalography (EEG) (Falkenstein et al., 2000; Gehring & Willoughby, 2002; Holroyd & Coles, 2002; Nieuwenhuis et al., 2001; Overbeek et al., 2005; Walsh & Anderson, 2012), skin conductance response (Crone et al., 2004), pupil dilation (Critchley et al., 2005), and in transient cardiac deceleration (Crone et al., 2003, 2006; Kastner et al., 2017; Somsen et al., 2000; van der Veen et al., 2004). Together, they have been suggested to constitute an internal readout of the performance monitoring mechanism, reflecting mismatches between an internal representation of the response or its predicted outcome, and the actual outcome (Jocham & Ullsperger, 2009; Ullsperger et al., 2014).

Cardiac deceleration in response to feedback has been extensively studied in the context of learning and decision‐making. The heart decelerates more in response to negative feedback after an error in performance (such as an erroneous choice), than in response to positive feedback, which elicits faster recovery (Crone et al., 2003, 2005; Somsen et al., 2000; van der Veen et al., 2004). This error‐related deceleration is initiated in anticipation of feedback and continues until after its presentation, and is likely to reflect error processing and the need for behavioral adjustment as part of the orienting response (Obrist, 1968; Pavlov, 1927; Sokolov, 1963). Notably, this response is only evident when feedback is valid—that is, when it carries reliable, usable information about performance (Crone et al., 2003; Groen et al., 2007; Mies et al., 2011). Feedback is considered valid or informative when it can be related to the nature of the stimulus—such as when the stimulus reliably predicts the response‐related outcome, and the feedback reflects the match or mismatch between the predicted and actual outcomes. Indeed, the strength of the phasic cardiac response to negative feedback has been found to correlate with the strength of the prediction error signal (derived from a reinforcement learning algorithm; Kastner et al., 2017), which alerts the learner to the need to adjust behavior.

It is noteworthy that during a learning task the monitoring mechanism precedes explicit awareness of the stimulus–response outcome contingencies. The error‐related cardiac deceleration response has been found to diminish as learning progresses, instead becoming more pronounced in anticipation of negative feedback than following it (Crone et al., 2003, 2004; Groen et al., 2007), or even emerging as soon as the stimulus predicting the negative outcome appears (Kastner et al., 2017). A similar point has been highlighted in the Somatic Marker Hypothesis, which originated from research showing that skin conductance response in a gambling task increased when participants pondered a choice from risky decks of cards, as opposed to safe decks, and preceded explicit awareness of the contingencies (Bechara et al., 1994; Poppa & Bechara, 2018). As such, autonomic performance monitoring appears to parallel or support the process of learning, preceding its behavioral manifestation in correct choices.

While error‐related cardiac deceleration appears reliably in instrumental learning paradigms when both the stimuli and the feedback are consciously presented (e.g., probabilistic instrumental learning in Kastner et al., 2017), the extent to which performance monitoring operates in this form of learning when the stimuli are presented unconsciously remains unclear. In an instrumental learning task, participants learn the associations between stimuli and their action‐related outcomes from feedback, and eventually learn to choose the stimuli associated with positive outcomes, while avoiding those associated with negative ones. In its unconscious version, all stimuli are presented outside of conscious awareness (e.g., with use of visual masking techniques), which precludes the formation of conscious associations between the stimuli and their outcomes for explicit performance monitoring.

In spite of this absence of explicit knowledge of stimulus–response outcome contingencies, some previous research has presented evidence that instrumental learning can occur without conscious awareness of the stimuli—subjects appeared to have learned to approach rewarding stimuli and avoid punishing ones, presented below the threshold of conscious awareness (Mastropasqua & Turatto, 2015; Pessiglione et al., 2008). This implies that the performance monitoring mechanism (i.e., comparing the actual outcome to the outcome expected from a given stimulus, albeit without explicit awareness of the cue‐outcome contingencies) must have been active. However, the capacity for complex forms of unconscious learning, including those involving adjustment of responses in the absence of cue or contingency awareness, has recently been disputed (Mertens & Engelhard, 2020; Travers et al., 2018), with evidence showing that instrumental learning fails when the absence of awareness is rigorously controlled (Reber et al., 2018; Skora et al., 2021). An absence of learning in those cases suggests that performance monitoring was impaired or absent. Hence, determining whether or not the autonomic signatures of performance monitoring are observable in this scenario can help to resolve the active debate around the feasibility of unconscious instrumental learning.

Importantly, in perceptual tasks, it appears that internal signatures of performance monitoring can manifest even in the absence of reportable awareness of the stimuli. Error‐related cardiac deceleration has been shown to occur in an unconscious stimulus discrimination task, where participants judged the orientation of a masked Gabor patch, albeit to a lesser extent than visible stimuli (Łukowska et al., 2018). Similarly, ERN has been shown to follow errors committed in response to stimuli presented below the awareness threshold (Pavone et al., 2009). While those are not instances of instrumental learning, they suggest that there could be cardiac activity indicative of differentiating between the stimuli before any behavioral manifestation of instrumental learning (i.e., adjustment of behavior). Error‐related cardiac deceleration should only be observed if the stimuli can be differentiated as rewarding or punishing, and the feedback reliably reflects the match or mismatch between their associated expected outcome and the actual outcome following from the action taken. Hence, such an effect could shed light on the viability of instrumental learning—the presence of error‐related cardiac deceleration would imply that the values of the unconsciously presented stimuli were successfully learned, regardless of behavioral evidence for learning manifested in correct choices.

The present study sought to directly answer the essential question whether autonomic performance monitoring is engaged in an unconscious instrumental conditioning paradigm. We employed a two‐stimulus, deterministic unconscious instrumental conditioning task (Skora et al., 2021, experiment 1), modeled on past paradigms (Pessiglione et al., 2008) and accompanied by a continuous measure of cardiac activity via an electrocardiogram (ECG) to assess the presence of error‐related heart rate deceleration. We sought to contrast learning and autonomic signatures of performance monitoring in conscious versus unconscious learning conditions. However, in order to maximize the potential for unconscious learning we wanted to select as simple a learning task as possible. This had the downside that an equivalent conscious task would have been too easy for participants, yielding an insufficient number of errors to analyze error‐related deceleration (verified by a pilot study). Fortunately, when engaged in the unconscious instrumental conditioning task participants exhibit transient moments of awareness on some trials, allowing them to occasionally discriminate the nature of the masked stimuli (see Skora et al., 2021). Thus, we were able to achieve the required comparison by contrasting aware and unaware trials from the same task and exploit the conscious trials to estimate an expected effect size for the unconscious condition.

The study has two potential outcomes, both of which would provide important insights. If performance monitoring is engaged in unconscious instrumental learning, we should observe its internal reflection as a pattern similar to that expected from conscious trials, albeit to a smaller extent. Such evidence, even in the absence of behavioral manifestation of learning, would suggest that, despite the complex nature of instrumental conditioning, people are able to acquire the knowledge of the stimulus nature (i.e., rewarding or punishing), without necessarily being able to act upon it instrumentally. This would shed light on the instrumental learning process and its relationship to consciousness. In contrast, an absence of autonomic signatures of performance monitoring in the unconscious trials would indicate an inability to acquire knowledge of the stimulus values and would strengthen the case against the feasibility of instrumental conditioning in the absence of conscious stimuli awareness in humans.

## 2 METHOD

### 2.1 Participants

Forty participants (25 females, 13 males, 2 participants did not report their gender) with a mean age of 25 years (*SD* = 3.27, range = 21–33; 7 participants did not report their age) were recruited for participation via the University of Sussex online recruitment system. Sample size was determined using the Bayesian Stopping Rule, with data collection continuing until a sensitive result was obtained in the learning task after conscious trials were excluded (see Section 4.1). All participated in exchange for course credit or £9.00. All participants reported having normal or corrected‐to‐normal vision, and no current or history of cardiac or neurological illness. Data for one participant was removed due to software malfunction, yielding a final sample of 39. Ethical approval was granted by the School of Psychology ethics committee at the University of Sussex, and the study was conducted in accordance with the Declaration of Helsinki.

### 2.2 Stimuli and materials

The experiment was conducted using Matlab 2018b (MathWorks, 2018), running Psychophysics Toolbox (Brainard, 1997). All stimuli were presented on a Samsung 2233RZ LCD monitor (1680 by 1050) with a refresh rate of 120 Hz, with the aim of ensuring fast and precise stimulus presentation in line with previous recommendations (Wang & Nikolić, 2011). The target stimuli included 6 neutral symbols obtained from Agathodaimon font in the main task, and two circular shapes in the perceptual discrimination task used for threshold finding. All were 180 × 180 pixels (7.45° visual angle) in size, and presented in light gray (RGB: 217, 217, 217) on a white background. The stimuli were forward and backward masked with black and white noise masks, again 180 × 180 pixels in size, comprising 50% white and 50% black pixel blocks 3 × 3 pixels wide. The forward and backward masks were generated afresh on each trial by randomly re‐scrambling the noise image. Low contrast cues and the type of mask were deliberately chosen in order to increase the duration of presentation without conscious awareness, following Scott et al. (2018). Responses were collected with a standard keyboard.

### 2.3 Electrocardiography

Electrocardiogram (ECG) was recorded for the duration of the main task using Biopac MP36, running Biopac Student Lab 3.7.7 (Biopac Systems, 2012), with a sampling rate of 500 Hz. The data were acquired using three disposable Ag/AgCl ECG electrodes, two placed below the left and right collarbones, and one on the left back, below the ribs.

### 2.4 Procedure

#### 2.4.1 Threshold setting

Participants were seated with their chin on a chin rest placed at 45 cm distance from the screen. Each session began with the determination of the threshold of visual awareness individually for each participant, using a masked perceptual discrimination task. Each trial began with a fixation cross (500 ms), followed by a mask (300 ms), a target cue (either a symmetrical circular shape or an asymmetrical circular shape, starting at 600 ms), and another mask (300 ms). After each sequence, participants were asked to determine whether the target cue was symmetrical or asymmetrical by pressing corresponding arrows. Next, they were asked to assess their level of confidence in that judgment, also using corresponding arrows (following Scott et al., 2018). They were explicitly instructed to press “some confidence” if they had confidence in their judgment, even a hunch, and ‘total guess’ only if they had no idea what the cue was, and were responding randomly. Each time a correct response was made with confidence, the display duration of the target cue was reduced by 50 ms on the following trial. When a duration of 100 ms was reached, or the first guess response was made, the display duration returned to the previous level (+50 ms), and subsequently reduced in 8.35 ms steps on the following trials, corresponding to a single screen refresh duration on a 120 Hz monitor. A reduction in exposure duration continued to be made after each non‐guess response but not after guess responses. This process continued until they indicated guessing on 5 consecutive trials, regardless of the accuracy of responses. The cue display duration in those trials was recorded as their individual unconscious threshold.

#### 2.4.2 Learning task

**FIGURE 1:**
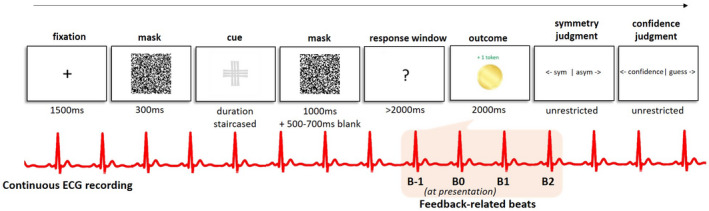
Unconscious instrumental conditioning task (main task). Chronological screenshots depict a single trial sequence, with durations in milliseconds. After cue presentation using forward‐backward masking, participants had 2 s to make a Go response with the spacebar, or refrain from responding (NoGo). Following the response, feedback was immediately displayed on the screen. In the example shown, a participant responded Go, which was a correct response for the cue presented, and was rewarded with one gold token. ECG was collected continuously throughout the task. For the analysis, beats surrounding feedback (the key event of interest) were extracted, where B0 is the first beat following the onset of feedback, B‐1 is the preceding beat, and B1 and B2 are the next beat and the second beat after, respectively (illustrated in a simplified fashion)

The main task was adapted from the unconscious instrumental conditioning task used previously (Pessiglione et al., 2008; Skora et al., 2021), in which participants make speeded go or no‐go responses to the masked cues. Here, each trial consisted of a fixation cross (1500 ms in order to record HR at baseline), forward mask (300 ms), target cue (display duration determined in the perceptual discrimination task), backward mask (1000 ms), and blank screen jitter (500–700 ms), followed by a decision prompt in the form of a question mark, during which participants had 2 s to make a response (see Figure 1, for task chronology). Pressing the space bar (Go) indicated a decision to take the risk, at which point the participant could win 1 token (golden token displayed on the screen) or lose 1 token (a red cross over the golden token displayed), depending on the type of cue presented between the masks. Not pressing the space bar (NoGo) indicated a safe choice, which always resulted in a null outcome (grayed‐out token displayed). Participants were incentivized to maximize their earnings in the task through a prize draw, with the number of entries contingent on the amount of tokens won. Feedback was displayed for 2000 ms immediately following the decision (or after the 2‐s decision prompt elapsed, in case of NoGo).

Following the Go or NoGo response on each trial, participants were asked to determine whether the masked stimulus was symmetrical or asymmetrical by pressing corresponding arrows, followed by a binary assessment of confidence (“some confidence” or “total guess”), in a manner identical to the threshold setting task. If they responded correctly with confidence three times in a row, cue display time was reduced by 8.35 ms, corresponding to single screen refresh duration.

The task included one block of 120 trials, consisting of 60 rewarding and 60 punishing trials (i.e., an equal number of trials with rewarding and punishing stimuli, associated with the corresponding outcome if approached in a 100% deterministic manner), presented in a randomized order. Two target cues from the pool of 6 were randomly assigned to be rewarding and punishing. The task reported here was conducted as part of a larger suite of measures, including two other, independent conditions of this task (performed in a randomized order block‐wise, with a new pair of stimuli assigned to each block without repetition), as well as an interoceptive accuracy task, performed last. Those measures will not be reported here, but the full task description can be found in Appendix 1. Further details including a description, stimuli, and the task code with instructions are available on the Open Science Framework at https://osf.io/ruxy3/.

## 3 DATA PRE‐PROCESSING

### 3.1 Exclusion criteria

Three participants who made only Go or only NoGo responses (e.g., due to a failure to understand the task) were excluded from all analyses. For the analysis of unaware trials, all individual trials where participants made correct symmetry judgment with confidence were marked as aware and excluded (16.74%). Nine participants who were aware on more than 25% of all trials were also excluded. This threshold was applied in order to minimize the risk that extensive conscious exposure might subsequently influence unconscious processing and thus inflate the estimate of unconscious‐only learning. This yielded a final sample of 27 for the unaware analysis. For the analysis of aware trials, all trials marked as aware in the previous step were included. All participants who made any number of aware judgments allowing to compute type I *d′* (i.e., both Hits and False Alarms were present; see Section 4.1. for detail) were included, yielding a sample of 24 for the aware analysis. Analysis using boxplot inspection revealed no other outliers.

### 3.2 Electrocardiography

Initial pre‐processing of the ECG data was done in Biopac Student Lab 3.7.7. The ECG data was filtered offline with a high‐pass filter (1 Hz), and R‐peaks of the QRS complex were detected for the length of the task. Heart rate (HR) in beats per minute (BPM) and interbeat intervals (IBI) were subsequently computed from the R‐R intervals. The data, complete with task event markers, were then exported into Matlab, where BPM and IBI were identified for each subject at each event (at baseline, cue presentation, response window, and feedback presentation), during the concurrent beat (B0—the first R‐peak to occur during the event), as well as one beat before (B‐1), and one (B1) and two beats (B2) following each event. Note that due to the focus on feedback‐related cardiac activity the analysis here will focus only on beats surrounding feedback events (for interested readers, we present the cardiac events surrounding stimulus presentation in the Supplementary Materials). Events where the average BPM deviated from the subject's mean by more than 3 *SD*s were excluded from analysis (as reduced or inflated values could reflect electrode displacement, excessive motion, or other artifacts). A total of 1154 events (0.67%) were removed (note that this is from a total of 4 beats computed around 9 events in the task). Two participants were excluded due to large unusable segments of data. Data was complete for all participants included in the behavioral analyses.

## 4 RESULTS

### 4.1 Evidence of learning: Performance

On average, participants were found to execute more Go responses (59%) than NoGo responses (41%), regardless of stimulus type. To account for this response bias, type I *d′* (a Signal Detection Theoretic measure of sensitivity to signal versus noise; Stanislaw & Todorov, 1999) was computed for the learning data, treating Go responses to rewarding cues as Hits, and Go responses to punishing cues as False Alarms. Where the resulting measure of sensitivity is significantly greater than zero, it can be taken as an approximation of successful learning (i.e., discrimination between the cues). Group‐level type I *d′* scores were entered into a one‐sample *t*‐test against 0. A Bayes Factor (B; Dienes, 2015a, Dienes, 2016) was computed for the difference, with the predictions of H1 (learning is present) modeled as a half‐normal distribution centred on 0, with an SD equal to 0.7 (the expected effect size if learning is present, estimated from Pessiglione et al., 2008). In line with the Bayesian Stopping Rule (Dienes, 2015b), data collection continued until a sensitive result was found in support of either H_0_ (absence of learning; by convention indicated by a B smaller than 1/3rd) or H_1_ (presence of learning; indicated by a B larger than 3). To indicate the robustness of Bayesian conclusions, a robustness region for the B was also computed, giving the range of scales that qualitatively support the same conclusion, i.e., evidence as insensitive (notated as RR_1/3<B<3_ [*x*1, *x*2]), as supporting H0 (RR_B<1/3_ [*x*1, *x*2]), or as supporting H1 (RR_B>3_ [*x*1, *x*2]), where *x*1 is the smallest SD that gives the same conclusion and *x*2 is the largest (see Dienes, 2019).

**FIGURE 2:**
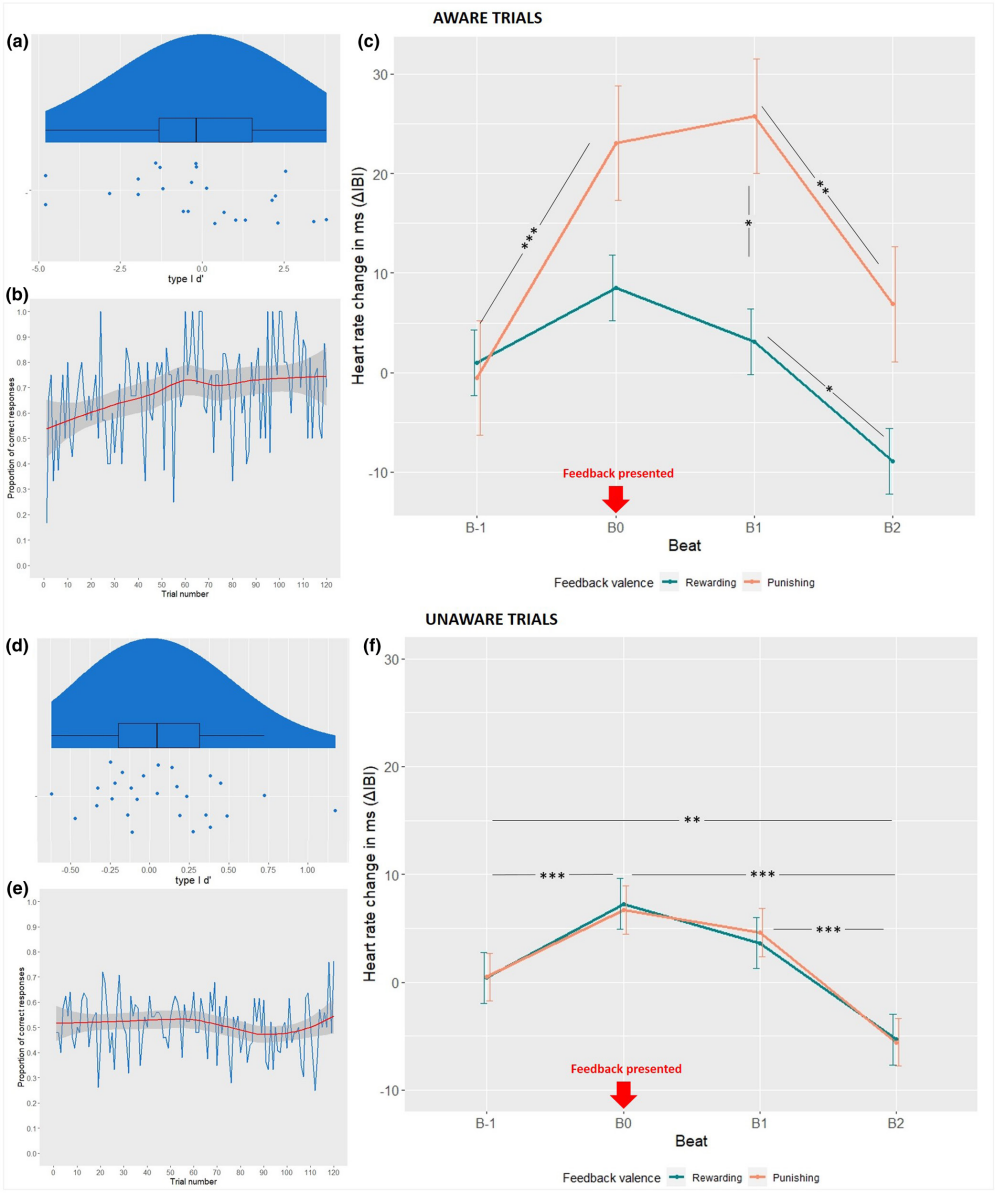
(a/d) Behavioral results; distribution of type I *d′* scores, with boxplots and individual data points, for aware (a) and unaware (d) trials. (b/e) Behavioral results; average proportions of correct responses (Go to rewarding and NoGo to punishing cues) across the length of the trial block, for aware (b) and unaware (e) trials. Ribbon represents a regression line (90% CI). In aware trials (top), participants' choices throughout the duration of the block show an upwards tendency, demonstrating learning. In unaware trials (bottom), participants' choices remained erratic and around the chance level, failing to display any evidence of learning the correct decisions. (c/f) Cardiac activity results; change in cardiac activity in ΔIBI (ms; ±1 *SEM*) in response to performance feedback (rewarding, punishing). A larger IBI indicates a longer time between each consecutive beat, reflecting cardiac deceleration. All beats are referenced to average IBI at B‐1 (1 beat before feedback presentation). B0 reflects the IBI measured at the point of feedback delivery. In aware trials (c), stars indicate significant difference between means of interest. In the unaware trials (f), stars indicate significant difference between means averaged across both feedback types due to no evidence for a main effect of feedback valence (*: <.05, **: <.01, ***: <.001)

When aware trials were excluded, in a one‐sample t‐test, the total *d′* was not significantly different from 0 (*M* = 0.07, *SE* = 0.08, *t*(26) = 0.97, *p* = .339, B_H(0,0.7)_ = 0.266, RR_B<1/3_ [0.6, Inf]), suggesting that participants were not able to learn the cue‐outcome associations. See Figure 2 for a graphical representation. A supporting analysis using binary logistic regression is available in the Supplementary Materials.

On aware trials only, where *d*′ was computable (i.e., both Hits and False Alarms were present), it was also not significantly different from 0, but the result was insensitive by the Bayesian criterion (*M =* −0.08, *SE* = 2.26, *t*(23) = −0.17, *p* = .865, B_H(0,0.7)_ = 0.945, RR_1/3<B<3_ [0, 6.9]. We attribute this to the large degree of variability in aware trials in our sample (5–102), where a small amount of trials may have been insufficient to learn the contingencies. For the sake of rigor, we decided against choosing an arbitrary threshold for inclusion of the aware trials.^1^ However, looking at participants who were marked as aware due to having over 25% of aware trials, the *d*′ was considerably higher (*M =* 0.92, *SE* = 1.78), but this was not significantly different from 0 and insensitive due to the very small sample of 8 (*t*(7) = 1.46, *p* = .188, B_H(0,0.7)_ = 1.087, RR_1/3>B>3_ [0, 9). Importantly, since the autonomic signatures of performance monitoring precede behavioral evidence of learning, the presence of learning in the aware trials is not a necessary precondition for observing the autonomic signatures of performance monitoring. Instead, it is the fact that the stimuli on those trials were consciously perceived in the first place.

### 4.2 Cardiac responses to rewarding versus punishing performance feedback

Following past research, we assessed whether the heart decelerates more following punishing performance feedback (i.e., after a Go response to a punishing stimulus) than following rewarding performance feedback (i.e., after a Go response to a positive stimulus), separately for both unaware and aware trials. For these analyses, all beats surrounding feedback presentation (B‐1, B0, B1, B2) were average‐referenced to the beat preceding feedback, B‐1. This reference was chosen over the fixation point in order to minimize the risk of contaminating the baseline with potential variations in cardiac activity at the earlier time points of the trial (e.g., stimulus presentation or action execution). For correspondence with previous papers, all following analyses use IBI as the index of heart rate change.

In order to assess whether the heart decelerates more following punishing than rewarding feedback, HR (indexed by ΔIBI in milliseconds) for both aware and unaware trials was submitted as a response variable into two separate linear mixed‐effects models, fit using the lme4 package (Bates et al., 2015) in R (R Core Team, 2018). Linear mixed‐effects models have an advantage over regular repeated‐measures ANOVA in that they are more robust to imbalances in the data (e.g., randomly missing values), and allow to incorporate each participant's individual baseline (random intercepts) and responsiveness to the manipulation (random slopes). The models included feedback valence (rewarding/punishing), beat (B‐1, B0, B1, B2) and their interaction as fixed effects (predictor variables). The random effects structure included subject‐specific random intercepts and random slopes for feedback valence. Note that this random effects formulation was used following the parsimonious approach given a singular fit under maximal specification (i.e., subject‐specific random intercepts and random slopes for the interaction of feedback valence and beat) (Matuschek et al., 2017). Treatment (dummy) coding was applied. The models were fit using maximum likelihood estimation. Note that only Go trials were used, as NoGo trials yielded no performance feedback and were not informative for task performance (they were construed as a safe choice or a pass).

#### 4.2.1 Aware trials

See Table 1 for regression coefficients from the aware model. Analysis of deviance on this model, conducted using the *car* package (Companion to Applied Regression; Fox & Weisberg, 2019) revealed a significant main effect of beat (*χ* ^2^ = 41.02, *df* = 3, *p* < .001), a main effect of feedback valence (*χ* ^2^ = 6.87, *df* = 1, *p* = .009), and an interaction between feedback valence and beat (*χ* ^2^ = 14.69, *df* = 3, *p* = .002). In line with past research, the results display a clear anticipatory deceleration (elongated IBI) before punishing feedback (B‐1 to B0), continuing after feedback has been delivered (B0 to B1), reflecting a reaction to feedback, and recovering by B2 (see Figure 2). For rewarding feedback, the deceleration was smaller in magnitude. The key contrast—magnitude of deceleration following feedback (B1)—shows a significant difference between rewarding and punishing feedback types (*M*_diff_ = 22.67, *SE* = 6.55, *p* = .019; for a full table of pairwise comparisons see the Supplementary Materials).

**TABLE 1:** Regression coefficients for the fixed effects from the linear mixed model on the aware trials (performance feedback events)

	Estimate (IBI)	*SE*	*df*	*t*‐Value	*p*
Intercept (B‐1:REW)	1.01	3.16	84.33	0.32	.751
PUN	−1.56	6.34	55.92	−0.25	.806
B0	7.50	3.87	1934.32	1.94	.053
B1	2.09	3.87	1934.32	0.54	.560
B2	−9.91	3.87	1934.32	−2.60	.011*
PUN:B0	16.12	6.55	1934.32	2.46	.014*
PUN:B1	24.26	6.55	1934.32	3.70	<.001***
PUN:B2	17.33	6.55	1934.32	2.64	.008**

*Note*: The intercept refers to B‐1 for rewarding feedback. *N* = 35, number of observations = 1984 (rewarding outcomes = 1292; punishing outcomes = 692).

Stars indicate significance levels at: *: <0.05, **: <0.01, ***: <0.001.

#### 4.2.2 Unaware trials

See Table 2 for regression coefficients from the unaware model. Analysis of deviance on this model, conducted using the *car* package, revealed a significant main effect of beat (*χ* ^2^ = 58.94, *df* = 3, *p* < .001), but no main effect of feedback valence (*χ* ^2^ = 0.01, *df* = 1, *p* = .968), and no interaction between feedback valence and beat (*χ* ^2^ = 0.22, *df* = 3, *p* = .975). As such, we found no support for cardiac differentiation between punishing and rewarding feedback in the unconscious conditioning task. Instead, cardiac deceleration (elongated IBI) was evident for both kinds of feedback (see Figure 2). Regardless of feedback valence, cardiac deceleration initiated upon feedback anticipation (B‐1 to B0) and continued until one beat after feedback presentation (B0 to B1), before recovering by the second beat.

**TABLE 2:** Regression coefficients for the fixed effects from the linear mixed model on the unaware trials (performance feedback events)

	Estimate (IBI)	*SE*	*df*	*t*‐Value	*p*
Intercept (B‐1:REW)	0.395	2.39	62.01	0.17	.869
PUN	0.074	2.50	147.19	0.03	.976
B0	6.871	2.40	6695.42	2.86	.004**
B1	3.241	2.40	6695.51	1.35	.178
B2	−5.725	2.40	6695.46	−2.38	.017*
PUN:B0	−0.641	3.42	6695.47	−0.19	.851
PUN:B1	0.893	3.42	6695.46	0.26	.798
PUN:B2	−0.310	3.42	6695.45	−0.09	.928

*Note*: The intercept refers to B‐1 for rewarding feedback. *N* = 27, number of observations = 6761 (rewarding outcomes = 3418; punishing outcomes = 3343).

Stars indicate significance levels at: *: <0.05, **: <0.01, ***: <0.001.

Our key question was whether there would be any evidence of autonomic signatures of performance monitoring in unconscious learning, marked by a differentiation in deceleration following errors (punishing feedback) in contrast to correct responses (rewarding feedback). In order to confirm the absence of this difference, we computed Bs for mean IBI differences at each point of interest surrounding feedback presentation (B‐1, B0, B1, B2), in the unaware trials. For the computation, the predictions of H1 (difference is present) was modeled as a half‐normal distribution centred on 0, with SDs corresponding to mean differences at each point found in the aware trials. The elongated IBI for punishing relative to rewarding feedback expected crucially at B1 was not evident (with sensitive Bs; see Table 3 and Figure 2; for a full table of pairwise comparisons see the Supplementary Materials).

**TABLE 3:** Model‐estimated mean differences between IBIs for rewarding and punishing feedback valence at each beat of interest surrounding the feedback (presented at B0), unaware trials

Beat	Estimated IBI (ms) mean difference (pun‐rew)	*SE*	*p*	B_H(0,Xms)_	RR
B‐1	0.0738	2.50	1.000	B_H(0,1.56)_ = 0.857	RR_1/3>B>3_ [0, 8]
B0	−0.5676	2.51	1.000	B_H(0,14.56ms)_ = 0.145^a^	RR_1/3<B_ [6.6, Inf]
B1	0.9468	2.50	.999	B_H(0,22.67ms)_ = 0.155^a^	RR_1/3<B_ [11.2, Inf]
B2	−0.2361	2.50	1.000	B_H(0,15.77)_ = 0.148^a^	RR_1/3<B_ [7.3, Inf]

*Note*: Bs computed using mean differences obtained in the aware trials of the present experiment.

^a^ Indicates a sensitive Bayes Factor.

## 5 DISCUSSION

Successful learning and decision‐making rely on efficient monitoring of performance, comparing rewarding or punishing outcomes of one's behavior and comparing them to expected outcomes. Simultaneously, performance monitoring is reflected internally, manifested in feedback‐related autonomic signatures. Among those signatures, evidence shows that the heart responds to negative (error‐related) feedback with a more pronounced and longer deceleration than to positive feedback (Crone et al., 2003, 2004; Groen et al., 2007; Kastner et al., 2017). While this cardiac deceleration had been observed in instrumental conditioning when stimuli are consciously perceived, and in simpler tasks when stimuli are absent from awareness, it was not known whether this mechanism could operate in unconscious instrumental learning. Establishing whether this mechanism can also operate in learning without conscious awareness of the stimuli can shed light on the feasibility of unconscious learning, and consequently, on the relationship between instrumental behavior and consciousness. Here, we examined the evidence for autonomic signatures of performance monitoring, as apparent from error‐related heart rate deceleration, during a masked instrumental learning task, and contrasted trials where the masking eliminated awareness of the stimulus with those where it did not. Evidence for the presence of such a signature in the absence of conscious stimulus awareness would indicate that people can acquire the knowledge of stimulus values, irrespective of whether they can apply such knowledge to adjust the their choices. The absence of this signature would however strengthen the evidence that instrumental learning cannot proceed without conscious awareness.

The physiological results show that the heart failed to differentiate between rewarding and punishing performance feedback when the stimulus was not consciously perceived. This result is in contrast to the robust differentiation found in the trials where participants were aware of the stimulus presented. In the presence of awareness the results were consistent with past evidence showing that the heart decelerates more in response to punishing feedback (indicative of an error) than to rewarding feedback (Crone et al., 2003, 2005; Kastner et al., 2017; van der Veen et al., 2004). When the stimuli were not consciously perceived, cardiac deceleration was evident for both types of feedback. An absence a differentiated response to rewarding and punishing feedback suggests that the feedback was not informative—it did not reflect a mismatch between the action‐driven outcome expected from a given stimulus and the outcome indicated by visual feedback. This, in turn, suggests that the masked stimulus was not processed to the extent allowing for integration with feedback and updating of stimulus values. In other words, stimulus identity was not predictive of the expected outcome, so any feedback signifying the actual outcome was rendered meaningless and uninformative. This was reflected in the autonomic signatures of performance monitoring as the absence of differentiation in cardiac activity between rewarding and punishing feedback.

The absence of autonomic differentiation between rewarding and punishing feedback in the unaware trials might superficially appear inconsistent with previously reported evidence of error‐related cardiac deceleration in the absence of stimulus awareness (e.g., Łukowska et al., 2018; though note that in their task no explicit performance feedback was provided—HR deceleration was found following an error in a perceptual judgment). This might suggest that the nature of the unconsciously presented stimulus, in non‐instrumental tasks, can be processed sufficiently to be reflected in the autonomic markers. If so, the crucial difference between those results might lie in the complexity of instrumental learning, where the stimulus only acquires value through the consequences which follow from acting upon it. In a simple perceptual judgment task, participants judge a feature of the stimulus as forced‐choice alternatives (e.g., a left‐/right‐tilted Gabor patch). In contrast, in the instrumental task used here, participants choose whether or not to deploy an action in response to a stimulus, and learn from the positive or negative outcomes of this action. If stimulus values are never learned, there are no expected outcomes associated with them, and so actual outcomes cannot be informatively compared to reflect on performance accuracy—consequently, the feedback is meaningless, and there is no autonomic activity to reflect the match or mismatch.

The behavioral results demonstrate that, without conscious awareness of the stimuli, participants were unable to learn the associations between stimuli predictive of reward or punishment and their corresponding outcomes in order to approach or avoid them appropriately. Trial‐by‐trial measures of cue awareness ensured that only truly unconscious trials were categorized as such, and Bayes Factors allowed us to assert that the null result obtained indicates a genuine absence of learning. This result supports the notion that the performance monitoring mechanism was not able to relate the actual outcome (feedback) to the outcome expected from the unconsciously perceived stimulus in order to guide the learning process and adjust choices. In contrast, the aware trials showed an increased ability to differentiate between the rewarding and punishing stimuli (although, due to their smaller number, without reaching sensitivity). This suggests that consciously perceiving the stimuli permitted the performance monitoring mechanism to monitor their values and compare them to feedback, which was reflected internally in the pronounced error‐related deceleration. This result suggests that autonomic signatures of performance monitoring might precede the learning process when the stimuli are consciously perceived. While there have been proposals that such autonomic activity directly drives behavior (e.g., SMH; Bechara et al., 1994), future research and finer‐grained analyses are needed to determine the extent to which such signals are causally implicated in driving learning and correct decisions.

The behavioral results corroborate the findings from our previous investigation of this topic (Skora et al., 2021), and supports the proposals that instrumental learning might require some degree of awareness (e.g., Reber et al., 2018). While there is evidence that some forms of simple associative learning can be achieved without stimulus awareness (Dupoux et al., 2008; Scott et al., 2018), there is limited evidence for unconscious learning in more complex scenarios, including learning more complex associations or across larger spatiotemporal intervals (Faivre et al., 2014; Travers et al., 2018). It has been proposed that conscious access might be necessary for higher‐order processes, including selective, goal‐oriented decision‐making (Baars, 2002; Dehaene & Naccache, 2001;Dehaene & Changeux, 2011; Lamme, 2006), and longer‐term behavioral adaptations (de Lange et al., 2011; Kunde et al., 2012; Reber et al., 2018; van Gaal et al., 2012). By these accounts, consciousness allows for information exchange between separate cognitive modules through long‐lasting, long‐range recurrent interactions between brain areas. Hence, it might be essential for flexible and lasting information processing strategies, such as those driving instrumental learning, where information must be integrated across multiple distinct events, from processing the stimulus and its expected value, deploying action, comparing the expected and actual outcome, and updating expected values. Because the effects of unconscious processing are short‐lived and local, preventing long‐range associations (Baars et al., 2003; Dehaene et al., 2001; Melloni et al., 2007), such extensive integration might simply not be possible.

Nonetheless, it is important to address the ways in which our task may have affected our ability to observe unconscious instrumental learning. Firstly, it is plausible that the timing of the task contributed to the failure to observe learning, as well as the absence of cardiac differentiation. Our task introduced a lengthy (around 2 s) gap between the stimulus and feedback, which may have disrupted the temporal stimulus‐feedback integration. However, we consider this unlikely, since previous evidence suggests that instrumental learning fails to manifest with similar, as well as shorter, stimulus‐outcome delays (Skora et al., 2021). Secondly, some of the task features, such as low stimulus contrast or masking intervals, may have prevented unconscious perception of the cue in the first place. Yet, similar stimulus contrast has been previously applied in tasks successfully demonstrating unconscious associative learning (e.g., Scott et al., 2018), suggesting that the contrast‐mask combination used here should not preclude unconscious perception of the stimuli. Thirdly, it is possible that unconscious instrumental learning may require considerably more trials to succeed, or other means of enhancing the memory consolidation, such as sleep (Klinzing et al., 2019). Both could be fruitful avenues for further research into the question of the feasibility of instrumental conditioning.

Future paradigms might also be able to investigate the finer‐grained features of performance monitoring in unconscious learning. Reliance on electrocardiography limits the insight into the different components of outcome‐related activity, such as differentiating the ERN (Hajcak et al., 2003) from the more generalized, surprise‐related feedback‐related negativity (FRN; Hauser et al., 2014). Exploring those could shed more light on the process and feasibility of internal and external performance monitoring in unconscious forms of learning.

Finally, previous research noted that HR measurements can be confounded by respiration rate, as respiration can affect the length of interbeat intervals (Berntson et al., 1993). Whether or not to correct for respiration in HR measurements is an ongoing debate (Laborde et al., 2017; Quintana & Heathers, 2014). In the present experiment, we did not record respiration rates, so any potential influences of breath on cardiac deceleration, whether sharing a common basis or a confounding variable (Thayer et al., 2011), cannot be assessed.

To summarize, the present study investigated the essential question whether autonomic signatures of performance monitoring are observable in unconscious instrumental conditioning. We found an absence of cardiac differentiation between rewarding and punishing feedback, demonstrating that performance monitoring is not engaged when the stimuli are not consciously perceived. In contrast, the expected differentiation, manifested as a heart rate deceleration following erroneous but not correct choices, was clearly observable when the stimuli were consciously perceived. We also replicated the recent results that participants are unable to learn instrumentally without conscious stimulus awareness. Together, those results strengthen the case against the feasibility of instrumental conditioning in the absence of conscious stimuli awareness. They provide strong support for the notion that, at least in humans, consciousness might be necessary for behavioral adaptations, especially where selective action is required.

## CONFLICT OF INTEREST

The authors report no conflict of interest.

## AUTHOR CONTRIBUTIONS

**Lina I Skora:** Conceptualization; data curation; formal analysis; investigation; methodology; project administration; visualization; writing – original draft; writing – review and editing. **James J. A. Livermore:** Formal analysis; writing – review and editing. **Federica Nisini:** Investigation; writing – review and editing. **Ryan B. Scott:** Methodology; resources; supervision; writing – original draft; writing – review and editing.

## Supporting information


**TABLE S1** Binary logistic regression coefficient estimates for aware trials
**TABLE S2** Binary logistic regression coefficient estimates for unaware trials
**TABLE S3** All pairwise comparisons of model‐estimated mean differences between IBIs (model reported in the main paper), aware trials. Tukey‐adjusted for multiple comparisons within subjects
**TABLE S4** All pairwise comparisons of model‐estimated mean differences between IBIs (model reported in the main paper), unaware trials. Tukey‐adjusted for multiple comparisons within subjects
**TABLE S5** Regression coefficients for the fixed effects from the linear mixed model on the aware trials (stimulus events). The intercept refers to B‐1 for rewarding feedback
**TABLE S6** Regression coefficients for the fixed effects from the linear mixed model on the unaware trials (stimulus events). The intercept refers to B‐1 for punishing feedback
**FIGURE S1** Change in cardiac activity in ΔIBI (ms; ±1 *SEM*) in response to rewarding and punishing stimuli for aware (left) and unaware (right) trials. All beats are referenced to average IBI at B‐1 (1 beat before stimulus presentation). B0 reflects the IBI measured at the point of stimulus presentation. Starts indicate significant differences between means averaged across both feedback types due to no evidence for a main effect of stimulus valence in either sample (*: <.05, **: <.01, ***: <.001)

## Footnotes

^1^
For reference, when applying a threshold for inclusion at a minimum of 20 available aware trials, the average aware *d′* was higher, as expected (*M* = 0.91, *SE* = 0.52, *t*(12) = 1.75, *p* = .106, B_H(0,0.7)_ = 2.94, RR_1/3>B>3_ [0, 14]).

## APPENDIX 1

The learning task reported in the article was conducted as a control condition of a larger within‐subjects experiment, alongside two other, independent conditions of the learning task, as well as an interoceptive accuracy task, performed last.

In the full experiment, we applied the unconscious instrumental learning task, reported in the main article, in three independent conditions with or without real‐time cardiac feedback. Participants' cardiac activity was measured throughout each condition with an electrocardiogram (ECG). Real‐time cardiac feedback was provided by measuring cardiac activity with a finger pulse oximeter, and playing back individual beeps in each condition: 1) Synchronous (beeps were played synchronously to the recorded heart rate), 2) Asynchronous (beeps were played asynchronously to the recorded heart rate, with a 1.5 s delay), 3) Control (no cardiac feedback was provided, participants performed the task in silence with no manipulations). This design aimed at investigating whether synchronous feedback would improve the ability to learn without stimulus awareness, as compared to asynchronous feedback and the control condition. The data reported in this article come from the control condition, with no embedded cardiac manipulation.

The order in which the conditions were performed was randomized for each participant, with self‐paced breaks in between. Each condition used a new set of stimuli as the positive and negative cues (stimuli were randomly assigned between conditions for each participant, without replacement). Importantly, the conditions were fully independent—in each condition participants had to learn new associations between the stimuli, their actions, and the outcomes. The instructions were provided at the beginning of the experimental session, and did not differ between the conditions of the learning task (i.e., participants performed the same task each time).

Following the three conditions of the learning task, individual interoceptive ability was measured with a heartbeat discrimination (Katkin & Whitehead) task, where participant were requested to determine whether 10‐s segments of real‐time cardiac feedback played back to them were synchronous or asynchronous to their actual heartbeat.

Further details including the full description, stimuli, and the task code with instructions are available on the Open Science Framework at https://osf.io/ruxy3/.
