# Supplementary material for: Awareness is required for autonomic performance monitoring in instrumental learning: Evidence from cardiac activity
Source: Psychophysiology. 2022 Mar 18;59(9):e14047. doi: 10.1111/psyp.14047 (PMC9541215; doi:10.1111/psyp.14047)
Supplement: Supplementary file 1 — TABLE S1 Binary logistic regression coefficient estimates for aware trials TABLE S2 Binary logistic regression coefficient estimates for unaware trials TABLE S3 All pairwise comparisons of model‐estimated mean differences between IBIs (model reported in the main paper), aware trials. Tukey‐adjusted for multiple comparisons within subjects TABLE S4 All pairwise comparisons of model‐estimated mean differences between IBIs (model reported in the main paper), unaware trials. Tukey‐adjusted for multiple comparisons within subjects TABLE S5 Regression coefficients for the fixed effects from the linear mixed model on the aware trials (stimulus events). The intercept refers to B‐1 for rewarding feedback TABLE S6 Regression coefficients for the fixed effects from the linear mixed model on the unaware trials (stimulus events). The intercept refers to B‐1 for punishing feedback FIGURE S1 Change in cardiac activity in ΔIBI (ms; ±1 SEM) in response to rewarding and punishing stimuli for aware (left) and unaware (right) trials. All beats are referenced to average IBI at B‐1 (1 beat before stimulus presentation). B0 reflects the IBI measured at the point of stimulus presentation. Starts indicate significant differences between means averaged across both feedback types due to no evidence for a main effect of stimulus valence in either sample (*: <.05, **: <.01, ***: <.001) [file PSYP-59-e14047-s001.docx]

**Supplementary materials**

**Awareness is required for autonomic performance monitoring in instrumental learning: Evidence from cardiac activity**

Lina I. Skora, James J. A. Livermore, Federica Nisini, & Ryan B. Scott

1. **Supporting analysis of learning performance**

A binary logistic regression was used to assess the evidence for learning over the course of the task block, i.e. the effect of incrementally increasing trials on the likelihood of correct versus incorrect response^[[1]](#footnote-1)^. Odds ratios were obtained by exponentiation of the coefficient estimates.

- 1. **Aware trials**

In aware trials, a unit increase in trial was associated with an increase in the odds of a correct response, but the effect was not statistically significant (see Table S1). The model was not statistically significant (χ^2^(1) = 0.56, p = .45), and did not explain any variance in correct choices (Nagelkerke R^2^ = 0%). This result is likely due to the small and uneven number of aware trials across participants (ranging between 5-102), which in many participants may have been insufficient to learn the contingencies (see main paper). This supports the *d*’ analysis reported in the paper.

*Table S1.* Binary logistic regression coefficient estimates for aware trials.

|  | **Estimate** | **Exp(Estimate)** | **SE** | **z-value** | **p-value** |
| --- | --- | --- | --- | --- | --- |
| Intercept | 0.563 | 1.756 | 0.217 | 2.593 | 0.010 ** |
| Trial | 0.002 | 1.002 | 0.002 | 0.752 | 0.452 |

- 1. **Unaware trials**

In unware trials, a unit increase in trial was associated with a decrease in the odds of a correct response, but the effect was not statistically significant (see Table S2). The model was not statistically significant (χ^2^(1) = 0.47, p = .49), and did not explain any variance in correct choices (Nagelkerke R^2^ = 0%). This result supports the d’ analysis reported in the paper.

*Table S2.* Binary logistic regression coefficient estimates for unaware trials.

|  | **Estimate** | **Exp(Estimate)** | **SE** | **z-value** | **p-value** |
| --- | --- | --- | --- | --- | --- |
| Intercept | 0.097 | 1.102 | 0.101 | 0.964 | 0.335 |
| Trial | -0.001 | 0.999 | 0.001 | -0.683 | 0.495 |

1. **Full pairwise comparisons for feedback-related cardiac activity**
   1. **Aware trials**

*Table S3.* All pairwise comparisons of model-estimated mean differences between IBIs (model reported in the main paper), aware trials. Tukey-adjusted for multiple comparisons within subjects.

|  | **Contrast** | | **Estimated IBI (ms) mean difference** | | ***SE*** | | ***p*** | |  |
| --- | --- | --- | --- | --- | --- | --- | --- | --- | --- |
| PUN-REW | | PUN B-1 – REW B-1 | | -1.56 | | 6.55 | | 1.000 | |
|  |  | PUN B0 – REW B0 | | 14.56 | | 6.55 | | 0.349 | |
|  |  | PUN B1 – REW B1 | | 22.67 | | 6.55 | | 0.019 | |
|  |  | PUN B2 – REW B2 | | 15.77 | | 6.55 | | 0.251 | |
| REW-REW | | REW B-1 – REW B0 | | -7.50 | | 3.88 | | 0.527 | |
|  |  | REW B-1 – REW B1 | | -2.09 | | 3.88 | | 0.999 | |
|  |  | REW B-1 – REW B2 | | 9.91 | | 3.88 | | 0.173 | |
|  |  | REW B0 – REW B1 | | 5.41 | | 3.88 | | 0.860 | |
|  |  | REW B0 – REW B2 | | 17.41 | | 3.88 | | <.001 | |
|  |  | REW B1 – REW B2 | | 11.99 | | 3.88 | | 0.042 | |
| PUN-PUN | | PUN B-1 – PUN B0 | | -23.64 | | 5.30 | | <.001 | |
|  |  | PUN B-1 – PUN B1 | | -26.31 | | 5.30 | | <.001 | |
|  |  | PUN B-1 – PUN B2 | | -7.42 | | 5.30 | | 0.857 | |
|  |  | PUN B0 – PUN B1 | | -2.69 | | 5.30 | | 0.999 | |
|  |  | PUN B0 – PUN B2 | | 16.20 | | 5.30 | | 0.047 | |
|  |  | PUN B1 – PUN B2 | | 18.89 | | 5.30 | | 0.009 | |

- 1. **Unaware trials**

*Table S4.* All pairwise comparisons of model-estimated mean differences between IBIs (model reported in the main paper), unaware trials. Tukey-adjusted for multiple comparisons within subjects.

|  | **Contrast** | | **Estimated IBI (ms) mean difference** | | ***SE*** | | ***p*** | |  |
| --- | --- | --- | --- | --- | --- | --- | --- | --- | --- |
| PUN-REW | | PUN B-1 – REW B-1 | | 0.0738 | | 2.50 | | 1.000 | |
|  |  | PUN B0 – REW B0 | | -0.5676 | | 2.51 | | 1.000 | |
|  |  | PUN B1 – REW B1 | | 0.9468 | | 2.50 | | 0.999 | |
|  |  | PUN B2 – REW B2 | | -0.2361 | | 2.50 | | 1.000 | |
| REW-REW | | REW B-1 – REW B0 | | -6.87 | | 2.40 | | 0.081 | |
|  |  | REW B-1 – REW B1 | | -3.2406 | | 2.40 | | 0.881 | |
|  |  | REW B-1 – REW B2 | | 5.7245 | | 2.40 | | 0.250 | |
|  |  | REW B0 – REW B1 | | 3.6301 | | 2.41 | | 0.803 | |
|  |  | REW B0 – REW B2 | | 2.5952 | | 2.41 | | <.001 | |
|  |  | REW B1 – REW B2 | | 8.9651 | | 2.41 | | 0.003 | |
| PUN-PUN | | PUN B-1 – PUN B0 | | -6.23 | | 2.43 | | 0.170 | |
|  |  | PUN B-1 – PUN B1 | | -4.11 | | 2.43 | | 0.692 | |
|  |  | PUN B-1 – PUN B2 | | 6.03 | | 2.43 | | 0.203 | |
|  |  | PUN B0 – PUN B1 | | 2.12 | | 2.43 | | 0.989 | |
|  |  | PUN B0 – PUN B2 | | 12.26 | | 2.43 | | <0.001 | |
|  |  | PUN B1 – PUN B2 | | 10.15 | | 2.43 | | <0.008 | |

1. **Cardiac activity in response to stimuli**

We additionally assessed whether cardiac activity differentiates between rewarding and punishing stimuli (i.e. those bringing a reward or punishment if approached). Similarly to the feedback analysis in the paper, this was done separately for both aware and unaware trials. For both, all beats surrounding stimulus presentation (B-1, B0, B1, B2) were average-referenced to the beat preceding the stimulus, B-1. The analysis uses IBI as an index of heart rate change.

HR (indexed by ΔIBI in milliseconds) for both aware and unaware trials was submitted as a response variable into two separate linear mixed-effects models, fit using the lme4 package (Bates, Mächler, Bolker, & Walker, 2015) in R (R Core Team, 2018); see paper for details. The models included stimulus valence (rewarding/punishing), beat (B-1, B0, B1, B2) and their interaction as fixed effects (predictor variables). The random effects structure included subject-specific random intercepts and random slopes for feedback valence. Note that this random effects formulation was used following the parsimonious approach given a singular fit under maximal specification (i.e. subject-specific random intercepts and random slopes for the interaction of feedback valence and beat) (Matuschek, Kliegl, Vasishth, Baayen, & Bates, 2017). Treatment (dummy) coding was applied. The models were fit using maximum likelihood estimation.

- 1. **Aware trials**

See *Table S3* for regression coefficients from the aware model. Analysis of deviance on this model, conducted using the *car* package (Companion to Applied Regression; Fox & Weisberg, 2019), revealed a significant main effect of beat (χ² = 18.22, *df* = 3, *p* < 0.001), but no main effect of stimulus valence (χ²  = 2.45, *df* = 1, *p* = 0.118), and no interaction between stimulus valence and beat (χ²  = 2.96, *df* = 3, *p* = 0.397). As such, we found no support for cardiac differentiation between punishing and rewarding stimuli when they were consciously perceived, despite visual inspection revealing a slight acceleration for punishing stimuli (see Figure 1S). The true effect may be obscured by a relatively small number of aware trials.

*Table S5*. Regression coefficients for the fixed effects from the linear mixed model on the aware trials (stimulus events). The intercept refers to B-1 for rewarding feedback.

|  | *Estimate (IBI)* | *Std. Error* | *df* | *t-value* | *p* |
| --- | --- | --- | --- | --- | --- |
| Intercept (B-1:REW) | -1.05 | 3.46 | 38.23 | -0.303 | 0.764 |
| PUN | -2.74 | 4.65 | 28.75 | -0.59 | 0.559 |
| B0 | 0.32 | 3.48 | 2868.13 | 0.09 | 0.926 |
| B1 | 4.99 | 3.48 | 2868.13 | 1.43 | 0.152 |
| B2 | 8.54 | 3.49 | 2868.29 | 2.45 | 0.015* |
| PUN:B0 | -3.44 | 5.08 | 2868.14 | -0.68 | 0.499 |
| PUN:B1 | -7.39 | 5.08 | 2868.16 | -1.45 | 0.146 |
| PUN:B2 | 0.22 | 5.09 | 2868.57 | 0.04 | 0.966 |

- 1. **Unaware trials.**

See *Table S4* for regression coefficients from the unaware model. Analysis of deviance on this model revealed a significant main effect of beat (χ² = 29.66, *df* = 3, *p* < 0.001), but no main effect of stimulus valence (χ²  = 0.01, *df* = 1, *p* = 0.960), and no interaction between stimulus valence and beat (χ²  = 0.08, *df* = 3, *p* = 0.994). As such, we found no support for cardiac differentiation between punishing and rewarding stimuli when they were unconsciously perceived. Instead, cardiac deceleration (elongated IBI) was evident for both kinds of stimuli (see *Figure S1*)*.*

*Table S6*. Regression coefficients for the fixed effects from the linear mixed model on the unaware trials (stimulus events). The intercept refers to B-1 for punishing feedback.

|  | *Estimate (IBI)* | *Std. Error* | *df* | *t-value* | *p* |
| --- | --- | --- | --- | --- | --- |
| Intercept (B-1:REW) | 0.25 | 2.12 | 51.41 | 0.12 | 0.906 |
| PUN | -0.17 | 2.28 | 98.95 | -0.08 | 0.940 |
| B0 | 0.49 | 1.85 | 11200.09 | 0.27 | 0.790 |
| B1 | 4.83 | 1.85 | 11200.13 | 2.61 | 0.009** |
| B2 | 5.90 | 1.85 | 11200.12 | 3.18 | 0.001** |
| PUN:B0 | 0.48 | 2.61 | 11200.16 | 0.18 | 0.855 |
| PUN:B1 | 0.12 | 2.61 | 11200.18 | 0.04 | 0.965 |
| PUN:B2 | -0.23 | 2.61 | 11200.19 | -0.09 | 0.929 |


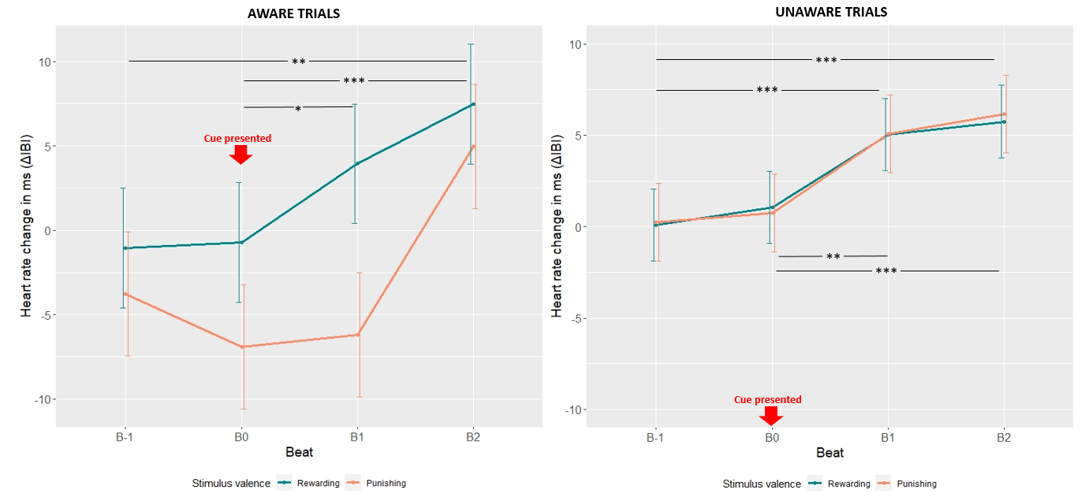


*Figure 1S.* Change in cardiac activity in ΔIBI (ms; +/- 1 SEM) in response to rewarding and punishing stimuli for aware (left) and unaware (right) trials. All beats are referenced to average IBI at B-1 (1 beat before stimulus presentation). B0 reflects the IBI measured at the point of stimulus presentation. Starts indicate significant differences between means averaged across both feedback types due to no evidence for a main effect of stimulus valence in either sample (*: <0.05, **: <0.01, ***: <0.001).

1. R notation used for both models: model = glm(correct ~ trial_in_block, data = data, family = binomial(link = "logit")) [↑](#footnote-ref-1)
